# Prevalence of* Helicobacter pylori cagA* and* iceA* Genes and Their Association with Gastrointestinal Diseases

**DOI:** 10.1155/2018/4809093

**Published:** 2018-04-05

**Authors:** Ashwak M. F. Abu-Taleb, Randa S. Abdelattef, Amina A. Abdel-Hady, Farida H. Omran, Lobna A. El-korashi, Hoda Abdel-aziz El-hady, Ahmed M. El-Gebaly

**Affiliations:** ^1^Medical Microbiology and Immunology Department, Faculty of Medicine, Zagazig University, Zagazig, Egypt; ^2^Internal Medicine Department, Faculty of Medicine, Zagazig University, Zagazig, Egypt; ^3^Tropical Medicine, Faculty of Medicine, Zagazig University, Zagazig, Egypt

## Abstract

*H. pylori* infection causes peptic ulcer, chronic gastritis, mucosa-associated lymphoid tissue lymphoma, and gastric carcinoma. It has several virulence factors such as cytotoxin-associated gene A(*cagA*) and the induced by contact with epithelium antigen (*iceA*). We aimed to explore the relationship between* cagA* and* iceA* of* H. pylori* and gastrointestinal diseases. One hundred and eighteen patients who attended Gastrointestinal Endoscopy Unit at Zagazig University Hospitals, Egypt, were included in this study. Two gastric biopsies were collected and evaluated by rapid urease test (RUT) and PCR.* cagA* and* iceA* genes were amplified by PCR. We found that 54 patients (45.76%) were positive by both RUT and PCR.* cagA* and* iceA* genes were present in 57.4% and 46.29% of the studied patients, respectively.* cagA* was the most prevalent gene in gastritis (33.3%) and peptic ulcer (68.7%).* iceA1/iceA2* positive genes were the most prevalent in gastric cancer (75%).* iceA1* gene was present in 38.7% of* cagA* positive cases, but* iceA2* gene was present in 45.2% of* cagA* positive cases.* iceA1/iceA2* positive genes were present in 29% of* cagA* positive cases. In conclusion,* cagA* and* iceA* genes could be used as markers for severe gastrointestinal diseases.* iceA* gene was strongly related to* cagA* gene.

## 1. Introduction

*Helicobacter pylori (H*.* pylori)* is a gram-negative microaerophilic spiral bacterium, which colonizes the gastric mucosa of approximately 50% of the human population in the world. A minority of the infected population suffer from chronic gastritis and peptic ulcer disease (PUD), and some even progress to gastric carcinoma (GC) and gastric mucosa-associated lymphoid tissue lymphoma [1]. Since 1994, the World Health Organization has classified it as class I carcinogen that the eradication of* H. pylori* can reduce the risk of gastric cancer [2].

Different virulence factors which play a role in the pathogenesis of the disease have been described, such as urease enzyme, flagella, adhesins, cytotoxin-associated gene* A (cagA)*, vacuolating cytotoxin A (*vacA*), and the induced by contact with epithelium (*iceA*) gene [3].

*H. pylori* enters the host stomach, and then it uses its urease enzyme to neutralize the acidic gastric condition at the start of infection. Flagella-intervened motility is then needed for* H. pylori* to move toward host gastric epithelium cells. After that, colonization and persistent infection are achieved by particular interactions between bacterial adhesins with host cell receptors. At last,* H. pylori* discharges multiple effector proteins/toxins, such as* cagA* and* vacA*, leading to host tissue damage. Moreover, the gastric epithelium layer secretes chemokines to start innate immunity and activate neutrophils, further leading to the formation of clinical diseases such as gastritis and ulcer [4].

“The* cagA*, a highly immunogenic protein, is encoded at one end of the cag pathogenicity island (cag PAI), which encodes the components to form the type IV secretion system (T4SS)” [5].* cagA* production has been supposed to be a measure of the virulence of* H. pylori* isolates.* cagA* was initially considered to act as a bacterial cytotoxin [6]. The* cagA* gene is reported to be found in more than half of the* H. pylori* isolates. It is known that* cagA* is associated with increased IL-8 production, nuclear factor-kB activation, mucosal inflammation, and development of PUD and GC [7].

The* cagA *was recognized as a cancer-associated factor long before its function was distinguished. Isolated strains from cancer patients frequently expressed* cagA*, while others from asymptomatic individuals or patients experiencing mild gastritis did not [8]. In addition to gastric carcinoma,* cagA* positive* H. pylori* is related to the development of gastric MALT lymphoma of B-cell origin. Eradication of* H. pylori* by antibiotics prompts regression of gastric MALT lymphoma in more than 75% of patients [9].

The* iceA* gene was identified in the* H. pylori *isolated from PUD and gastritis patients. There are at least two alleles of* iceA*,* iceA1*, and* iceA2* [10]. The expression of* iceA1* was upregulated on contact between* H. pylori* and human epithelial cells. The* iceA1* genotype was associated with enhanced mucosal IL-8 expression and acute antral inflammation. Furthermore, it was shown that adherence to gastric epithelial cells in vitro stimulates* iceA1* transcription [11]. Several studies suggest an association of the* iceA1* variant and PUD [12]. On the other hand,* iceA2* has no homology to known genes, and the function of the* iceA2* product remains vague in spite of the fact that this allele is associated with asymptomatic gastritis and nonulcer dyspepsia [12].

The* vacA* is a virulence factor present in nearly half of* H. pylori* isolates encoding the vacuolating cytotoxin in various mammalian cell lines in vitro. The* H. pylori* isolates are classified according to the presence of different families of* vacA* signal sequences* (s1a, s1b, s2)* and middle region alleles* (m1, m2)* [13]. Consistent with in vitro results, studies in the Middle East, Africa, and Western countries have revealed that individuals infected with* vacA s1* or* m1 H. pylori *strains have an elevated risk of peptic ulcer or gastric cancer compared with individuals infected with* s2* or* m2* strains [14]. On the other hand, in East Asia, as most strains are* vacA* s1, the type of “s” region cannot clarify the differences in pathogenesis. In turn, the “m” region in East Asia shows variations suggesting that it may play a role in the regional difference [15].

*dupA* (Duodenal ulcer promoting gene) may enhance duodenal ulceration and/or diminish gastric cancer development in some populations [16].* dupA* product stimulates the production of IL-8 and -12 from the gastric mucosa of the antrum in vivo and from gastric epithelial cells in vitro as well [17]. It can be considered as a disease-specific virulence marker even in East Asian nations such as Japan and South Korea [18]. Furthermore, an investigation also revealed that the presence of* dupA* was significantly associated with eradication failure [19].

Various studies have been conducted to demonstrate the relation between different virulence genes of* H. pylori *and the severity of gastrointestinal diseases. Most of the previous studies investigated* cagA* and* vacA* genes [20, 21]. In the current study, we explored the relation between* cagA* and* iceA* genes and severe gastrointestinal diseases as a continuation of the previous studies in Zagazig University Hospitals, Egypt, where* H. pylori* prevalence is expected to be high.

## 2. Methods

### 2.1. Study Setting

This study was conducted in Immunology Research and Molecular Biology Laboratories in the Microbiology and Immunology Department, Gastrointestinal Endoscopy Unit at Zagazig University Hospitals, and Scientific and Medical Research Center of Zagazig University, Faculty of Medicine, Zagazig University, Egypt, from January 2016 to May 2017.

### 2.2. Study Design

This is a cross-sectional study.

### 2.3. Study Participants

One hundred and eighteen patients were enrolled in this study by systematic random sample. They attended Gastrointestinal Endoscopy Unit at Zagazig University Hospitals, Egypt, for diagnostic endoscopy suffering from upper GIT symptoms or for any other diagnostic purposes or patients with previously diagnosed gastric carcinoma attending for follow-up endoscopy.

Careful history was taken from all subjects as regards age, sex, symptoms they suffer from, medications, previous endoscopy or operations in the stomach, and other extra GIT diseases. Subjects, who were less than 18 years old and received antimicrobial therapy, H_2_-receptor blockers, proton-pump inhibitors, and nonsteroidal anti-inflammatory drugs 2 months prior to endoscopy were excluded from the study. The endoscopic findings of each patient were classified into four categories: gastritis, peptic ulcer, gastric cancer, and mixed lesions (combinations between ulcer, gastritis, and polyp or mass).

### 2.4. Ethics

Approval for the study was provided by Microbiology and Immunology Department Committee, Ethical Committee at Faculty of Medicine and Institutional Review Board (IRB). The ethical committee number is ZU-IRB: 22062-24-8-2015. All participants were informed about the nature and the purpose of the study and written informed consent was obtained.

### 2.5. Diagnosis of* H. pylori* Infection

For every patient, two biopsy specimens were taken from the antrum and fundus using a disinfected endoscope; one was examined by RUT, and the other one was placed in 0.1 ml of sterile saline solution and was stored in −80°C for DNA extraction and PCR [22].

- RUT was done by using commercial paper RUT according to the manufacturer's protocol (HelicotecUT® Plus; Catalog number HUP01, Strong Biotech Corporation, Taiwan). The biopsy specimen was transferred onto the test paper with the applicator included in the test kit. Color changes were observed within one hour.
- DNA extraction and PCR amplification:
  - DNA was extracted from biopsies using the genomic DNA purification system according to the manufacturer's instructions (QIAamp® DNA Mini kit; catalog number 51304, QIAGEN, Germany) and stored at −20°C until analysis.
  - A sequence of 294 bp in the ureC (glmM) gene was amplified by PCR (Maxime PCR Premix Kit (i-Taq), catalog number 25025, iNTRON Biotechnology, Korea). Maxime PCR Premix (i-Taq) beads were designed as a premixed format, freeze-dried into a pellet. They were kept at −20°C. When reconstituted, each bead contained 2.5 U of i-Taq DNA polymerase (from thermos thermophilus HB7), 200 *μ*M of each dNTPs, 10 mM Tris-HCl (pH 9.0), 50 mM KCL, 1.5 mM Mgcl2, and 1x gel loading dye. It had every component for PCR, so PCR was done by adding a template DNA, primer set, and distilled water. It had gel loading buffer (1x) to do electrophoresis, so we did gel loading without any treatment. Primer pair used for ureC amplification was shown in Table 1. The amplification was carried out in a thermal cycler (Veriti® 96-Well Thermal Cycler, Applied Biosystems, Singapore) according to the following program: an initial denaturation step at 95°C for 10 min, followed by 35 cycles of denaturation at 95°C for 30 s, annealing at 55°C for 1 min, and a final extension step at 72°C for 5 min.
  - Amplified PCR products were resolved by agarose gel electrophoresis (5 V/60 min) using 1,5% agarose in Tris Acetate-EDTA (TAE) buffer containing 0.5 ug/mL of ethidium bromide. Molecular size ladder of 100 bp (Roche, Lewes, East Sussex) was used to determine the size of the bands. The gel was viewed and photographed over the UV transilluminator at 320 nm.

### 2.6. Detection of Virulence Genes

All DNA extracts of positive cases of both RUT and PCR tests were subjected to molecular detection of virulence genes (*cagA, iceA1*, and* iceA2*) [23]. These genes were amplified by PCR (Maxime PCR Premix Kit (*i*-Taq), catalog number 25025, iNTRON Biotechnology, Korea). Primer pair used was shown in Table 1.

The amplification was carried out in a thermal cycler as previously mentioned in ureC (glmM) gene but the annealing temperature was 55°C for* cagA*, 56°C for* iceA1*, and 50°C for* iceA2*.

### 2.7. Statistical Analysis

Data were collected and coded, and all analyses were performed using Statistical Package for the Social Sciences software (SPSS version 20, Inc., Chicago, IL, USA.). Data were entered as variables, represented by tables.

A Chi-square test and Fisher's exact test were used to assess the association among the genotypes and between specific genotypes and upper gastrointestinal diseases. Mann–Whitney *U* test and *t*- test were used for calculation of mean difference between different groups. All analyses were 2-tailed. Results were considered statistically significant when *P* (probability) values were equal to or less than 0.05 at confidence interval (CI) 95%.

## 3. Results

### 3.1. Diagnosis of* H. pylori* Infection

**Figure 1:**
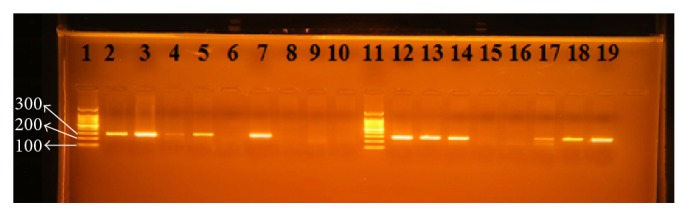
PCR products for* H. pylori* with glmM (ureC) gene-based primers. The product size is 294 bp. Lanes 1 and 11 are ladders. Lanes 2–10 and 12–19 are patients' biopsy samples.

RUT results were observed within a few minutes up to 1 hour. If the test paper changes color to pink or red, the test of* H. pylori* is positive. If it remains yellow in color, then the test is negative. Positive PCR results were observed on agarose gel as a band with 294 bp in size for glmM gene (Figure 1). There were 54 patients positive for* H. pylori* infection by both RUT and PCR.

### 3.2. Detection of Virulence Genes and Their Relation to Clinical Status

**Figure 2:**
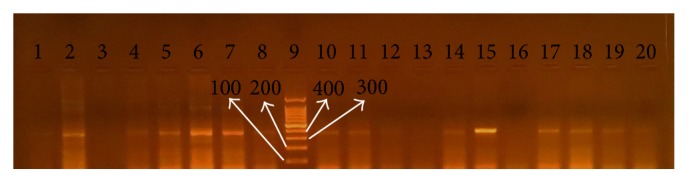
PCR products for* H. pylori* with* cagA* gene-based primers. The product size is 400 bp. Lane 9 is a ladder. Lanes 1–8 and 10–20 are patients' biopsy samples.

**Figure 3:**
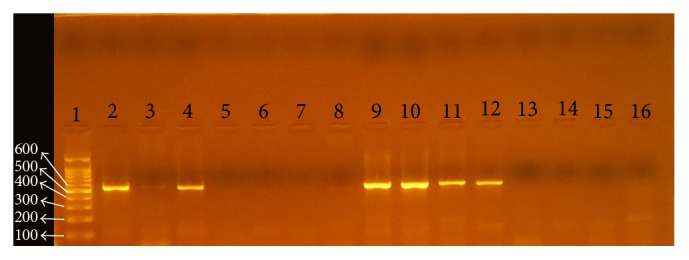
PCR products for* H. pylori* with* iceA1* gene-based primers. The product size is 558 bp. Lane 1 is a ladder. Lanes 2–16 are patients' biopsy samples.

**Figure 4:**
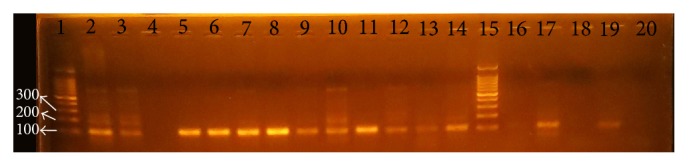
PCR products for* H. pylori* with* iceA2 *gene-based primers. The product size is 120 bp. Lanes 1 and 15 are ladders. Lanes 2–14 and 16–20 are patients' biopsy samples.

*cagA* and* iceA* genes were visualized under ultraviolet light as shown in Figures 2, 3, and 4, respectively. Analysis of the detected virulence genes revealed that there were 31 (57.4%)* cagA* positive strains and 25 (46.29%)* iceA* positive strains among the 54 positive* H. pylori* patients. Meanwhile,* iceA1* positive strains were six (11.11%) and* iceA2* positive strains were six (11.11%) as well. Thirteen (24.07%)* iceA1/iceA2* positive strains were identified among the 54 positive* H. pylori* patients.

There was statistically significant difference in the relation between clinical status by endoscope and different genes of* H. pylori* (*P* ≤ 0.05) as shown in Table 2. Among patients with gastritis, 33.3% of patient were* cagA* positive and about 68.7% of patients with peptic ulcer were* cagA* positive. Fifty percent of patients with gastric cancer were* cagA* positive. Meanwhile, all patients with mixed lesions were* cagA* positive.

Among patients with gastritis, about 16.7% of patients were* iceA1/A2* positive. The percentages of* iceA1*,* iceA2*, and* iceA1/A2* positive in peptic ulcer patients were 6.25%, 12.5%, and 18.8%, respectively. Meanwhile, in gastric cancer patients* iceA1/A2* positive genotype was the most prevalent (75%). Among patients with mixed lesions,* iceA1* and* iceA1/A2* positive were the most prevalent (30%) (Table 2).

*(i) Association between cagA and iceA Genes. *There was statistical significant association between* cagA* and* iceA* genes (*P* ≤ 0.05). IceA1 was present in 12 cases out of 31* cagA* positive cases (38.7%). On the other hand,* iceA1* was present in 7 cases out of 23* cagA* negative cases (30.4%).* iceA2* was present in 14 cases out of 31* cagA* positive cases (45.2%). Meanwhile,* iceA2* was present in 7 cases out of 23* cagA* negative cases (30.4%). As regards* iceA1/A2*+, they were present in 9 cases out of 31* cagA* positive cases (29%). However,* iceA1/A2* was present in 15 cases out of 23* cagA* negative cases (65.2%) (Table 3).

## 4. Discussion

*H. pylori *is a standout among the most widely recognized infectious agents around the world, and roughly half of the world's population is estimated to be infected [24]. Multiple diagnostic techniques are developed to detect* H. pylori *infection and are divided into two groups of invasive and noninvasive methods according to the necessity of endoscopic biopsy [25]. Several studies considered that one invasive test could be used for diagnosis of* H. pylori* infection and confirmation of* H. pylori* eradication after treatment [26, 27]. On the other hand, other studies considered that the combination of two positive invasive tests is required for diagnosis of* H. pylori* infection [28]. The present study used the combination of RUT and PCR for diagnosis of* H. pylori* infection.

Different genotypes of* H. pylori* produce various virulence factors. Urease enzyme, adhesins,* cagA*, and* vacA* are conclusively associated with severe gastroduodenal diseases. Some other virulence genes have been found, one of which is* iceA*, which is independent of* cagA* and* vacA *[29].

The present study has focused on characterizing the virulence genes of* H. pylori* from gastric biopsy specimens from patients with upper gastrointestinal diseases and their relationship with clinical status.* H. pylori* was analyzed for the presence of the genes for* cagA* and* iceA*.

Our study revealed that* cagA* gene was present in 57.4% of the studied subjects. However, several studies reported different percentages of* cagA* gene in different countries [22, 30–32].

In Egypt, several studies investigated the prevalence of* cagA* and they reported variable results [20, 33, 34]. Amer and her colleagues (2013) reported high prevalence of* cagA* gene (65%) [20]. Moreover, Said Essa and his colleagues (2008) reported that 62.2% of* H. pylori* infected patients were* cagA* positive [33]. On the other hand, El-Shenawy and his colleagues (2017) reported low prevalence of* cagA* gene (26.6%) [34]. This could be attributed to different sample sizes, different socioeconomic status, and living conditions of the studied patients.

Interestingly, our study found that* cagA* was present in 68.7% of peptic ulcer patients, 50% of patients with gastric carcinoma, 33.3% of patients with gastritis, and all patients with mixed lesions (100%). Likewise, several studies reported that* cagA* was more prevalent in peptic ulcer and gastric carcinoma than gastritis [31, 35]. Meanwhile, Kadi and her colleagues (2014) observed that* cagA* gene was more prevalent in patients with gastritis than peptic ulcer (85% and 77%, resp.) [32]. In the same context, Feliciano and his colleagues (2015) found no association between* cagA* gene and peptic ulcer, which could be influenced by the small number of patients studied with this pathology [36].

The present study reported that* iceA* was present in 46.29% of the studied patients. The percentages of* iceA1 *and* iceA2* among the studied subjects were the same (11.11%). Other studies investigated the prevalence of* iceA* and reported different percentages [31, 32]. In Egypt, El-Shenawy and his colleagues (2017) reported that* iceA* percentage was 38.8% [34]. This could be attributed to different sample sizes and different populations.

The relationship between* H. pylori iceA* and clinical outcomes is controversial. Our study revealed that* iceA1* gene was present in 30% of patients with mixed lesions, 8.3% of patients with gastritis, and 6.25% of peptic ulcer patients. Meanwhile,* iceA2* gene was less prevalent in patients with mixed lesions (10%) and more prevalent in gastritis and peptic ulcer (12.5%). None of patients with gastric carcinoma expressed either* iceA1* or* iceA2* gene alone. surprisingly,* iceA1/A2* positive cases were more prevalent in patients with gastric carcinoma (75%). This finding indicates that patients harboring both alleles of* iceA (iceA1/A2) *are at high risk of developing gastric carcinoma.* iceA1/A2* positive cases were less prevalent in gastritis, peptic ulcer, and mixed lesions (16.7%, 18.8%, and 30%, resp.)

In the same context, Huang and his colleagues (2016) demonstrated that the prevalence of* iceA1* significantly increased the risk of peptic ulcer compared with gastritis [1].

Our findings agreed with Huang and his colleagues (2016) as regards gastric carcinoma [1]. They found no significant risk association between* iceA1* status and gastric carcinoma in any country population, possibly due to the relatively small sample size of gastric carcinoma cases compared to peptic ulcer or gastritis. Surprisingly, Wei and his colleagues (2012) have found that only* iceA1 *gene had a statistically significant association with gastric cancer [31].

On the other hand, Feliciano and his colleagues (2015) did not agree with our finding as regards* iceA2* [36]. They found an association between* H. pylori* strains harboring the* iceA2* allele in patients with nonulcer disease. This behavior has also been described in Europe, Saudi Arabia, and Turkey [12]. However, Huang and his colleagues (2016) revealed no significant association between* iceA2* and clinical outcomes [1].

The current study reported a statistical significant association between* cagA* and* iceA* genes, (*P* ≤ 0.05).* iceA1* gene was present in 38.7%* cagA* positive cases, but* iceA2* gene was present in 45.2%* cagA* positive cases. As regards* iceA1/A2*, they were present in 29% of* cagA* positive cases. These findings were in agreement with several studies [31, 32, 37]. These findings clarify that* cagA* gene could be considered a predictor for the presence of* iceA* gene.

## 5. Conclusion

The present study showed that* H. pylori* virulence genes (*cagA* and* iceA*) were prevalent among patients with upper gastrointestinal diseases. The* cagA*+ was associated with peptic ulcer and mixed lesions. Interestingly,* iceA1/A2+ *was associated with increased risk of gastric cancer. Meanwhile,* iceA1*+ was more prevalent in patients with mixed lesions and* iceA2*+ was more prevalent in patients with gastritis and peptic ulcer. Therefore, these genes could be used as markers for severe upper gastrointestinal diseases. The* iceA* gene was significantly related to* cagA* gene.

## Figures and Tables

**Table 1:** Primer sequences used in this study.

Gene	Primer sequence	Size (bp)
*UreC (glmM)*	Forward: 5′- AA GCTTTTAGGGTGTTAGGGGTTT -3′	294
	Reverse: 5′- AAGCTTACTTTCTAACACTAACGC -3′	

*cagA*	Forward: 5′- AATACACCAACGCCTCCAAG -3′	400
	Reverse: 5′- TTGTTGGCGCTTGCTCTC -3′	

*iceA1*	Forward: 5′- CGTTGGGTAAGCGTTACAGAATTT -3′	558
	Reverse: 5′- TCATTGTATATCCTATCATTACAAG -3′	

*iceA2*	Forward: 5′- GTTGTCGTTGTTTTAATGAA -3′	120
	Reverse: 5′- GTCTTAAACCCCACGATTAAA -3′	

**Table 2:** Relation between clinical status by endoscope of *H. pylori* infection and different virulence genes.

	Clinical status by endoscope								Test of significance	*P* value
	Gastritis (*N* = 50)		peptic ulcer (*n* = 27)		Gastric cancer (*n* = 4)		Mixed lesion (*n* = 9)			
	*N*	%	*N*	%	*N*	%	*N*	%		
*cag A*										
* cag A+*	22	44.0	*18*	*66.7*	*2*	*50.0*	*9*	*100.0*	Fisher's exact test 178.93	<*0.001*^*∗*^
* cag A−*	28	56.0	9	33.3	2	50.0	0	0.0		
*ice A*										
* ice A1+*	*5*	10.0	2	7.4	0	0.0	3	33.3	Fisher's exact test 175.54	<*0.001*^*∗*^
* ice A2+*	*7*	14.0	3	11.1	0	0.0	2	22.2		
* ice A1∕A2+*	*11*	22.0	5	18.5	3	75.0	3	33.3		
* ice A−*	*27*	54.0	17	62.9	1	25.0	1	11.2		

*N*: number, %: percentage, *P* of Fisher's exact test, ^*∗*^*P* ≤ 0.05.

**Table 3:** Association between *cagA* and *IceA* genes.

	*cag A+* (*n* = 51)		*cagA−* (*n* = 67)		Test of significance, Fisher's exact test	*P* value
	*N*	%	*N*	%		
*ice A1*						
* iceA1+*	*16*	*31.4*	*15*	*22.4*	*161.94*	<*0.001*^*∗*^
* iceA1−*	35	*68.6*	*52*	*77.6*		
*ice A2*						
* iceA2+*	20	39.2	*15*	*22.4*	*164.54*	<*0.001*^*∗*^
* iceA2−*	31	60.8	*52*	*77.6*		
*ice A1 ∕A2*						
* ice A1 ∕A2+*	12	54.5	39	*40.6*	*162.39*	<*0.001*^*∗*^
* ice A1 ∕A2−*	10	45.5	57	*59.4*		

## References

[1] Huang X., Deng Z., Zhang Q., Li W., Wang B., Li M. (2016). Relationship between the iceA gene of Helicobacter pylori and clinical outcomes. *Therapeutics and Clinical Risk Management*.

[2] Kusters J. G., van Vliet A. H. M., Kuipers E. J. (2006). Pathogenesis of *Helicobacter pylori* infection. *Clinical Microbiology Reviews*.

[3] Yamaoka Y. (2010). Mechanisms of disease: *Helicobacter pylori* virulence factors. *Nature Reviews Gastroenterology & Hepatology*.

[4] Kao C.-Y., Sheu B.-S., Wu J.-J. (2016). Helicobacter pylori infection: An overview of bacterial virulence factors and pathogenesis. *Biomedical Journal*.

[5] Tegtmeyer N., Wessler S., Backert S. (2011). Role of the *cag*-pathogenicity island encoded type IV secretion system in *Helicobacter pylori* pathogenesis. *FEBS Journal*.

[6] Hatakeyama M. (2017). Structure and function of helicobacter pylori caga, the first-identified bacterial protein involved in human cancer. *Proceedings of the Japan Academy Series B: Physical and Biological Sciences*.

[7] Suzuki R., Shiota S., Yamaoka Y. (2012). Molecular epidemiology, population genetics, and pathogenic role of *Helicobacter pylori*. *Infection, Genetics and Evolution*.

[8] Stein M., Ruggiero P., Rappuoli R., Bagnoli F. (2013). *Helicobacter pylori* CagA: from pathogenic mechanisms to its use as an anti-cancer vaccine. *Frontiers in Immunology*.

[9] Ferreri A. J. M., Govi S., Ponzoni M. (2013). The role of Helicobacter pylori eradication in the treatment of diffuse large B-cell and marginal zone lymphomas of the stomach. *Current Opinion in Oncology*.

[10] Yakoob J., Abbas Z., Khan R. (2015). Helicobacter pylori: Correlation of the virulence marker iceA allele with clinical outcome in a high prevalence area. *British Journal of Biomedical Science*.

[11] Shiota S., Suzuki R., Yamaoka Y. (2013). The significance of virulence factors in *Helicobacter pylori*. *Journal of Digestive Diseases*.

[12] Amjad N., Osman H. A., Razak N. A., Kassian J., Din J., Abdullah N. B. (2010). Clinical significance of *Helicobacter pylori cagA* and *iceA* genotype status. *World Journal of Gastroenterology*.

[13] Isomoto H., Moss J., Hirayama T. (2010). Pleiotropic actions of Helicobacter pylori vacuolating cytotoxin, VacA. *The Tohoku Journal of Experimental Medicine*.

[14] Sugimoto M., Zali M. R., Yamaoka Y. (2009). The association of vacA genotypes and Helicobacter pylori-related gastroduodenal diseases in the Middle East. *European Journal of Clinical Microbiology & Infectious Diseases*.

[15] Roesler B. M., Rabelo-Gonçalves E. M. A., Zeitune J. M. R. (2014). Virulence factors of helicobacter pylori: A review. *Clinical Medicine Insights: Gastroenterology*.

[16] Hagymási K., Tulassay Z. (2014). Helicobacter pylori infection: new pathogenetic and clinical aspects. *World Journal of Gastroenterology*.

[17] Yamaoka Y. (2012). Pathogenesis of *Helicobacter pylori*-related gastroduodenal diseases from molecular epidemiological studies. *Gastroenterology Research and Practice*.

[18] Lu H., Hsu P.-I., Graham D. Y., Yamaoka Y. (2005). Duodenal ulcer promoting gene of Helicobacter pylori. *Gastroenterology*.

[19] Shiota S., Nguyen L. T., Murakami K. (2012). Association of helicobacter pylori dupA with the failure of primary eradication. *Journal of Clinical Gastroenterology*.

[20] Amer F. A., El-Sokkary R. H., Elahmady M. (2013). Helicobacter pylori genotypes among patients in a university hospital in Egypt: identifying the determinants of disease severity. *Journal of Microbiology and Infectious Diseases*.

[21] Aziz F., Chen X., Yang X., Yan Q. (2014). Prevalence and correlation with clinical diseases of *Helicobacter pylori* cagA and vacA genotype among gastric patients from Northeast China. *BioMed Research International*.

[22] Souod N., Kargar M., Doosti A., Ranjbar R., Sarshar M. (2013). Genetic analysis of cagA and vacA genes in helicobacter pylori isolates and their relationship with gastroduodenal diseases in the west of Iran. *Iranian Red Crescent Medical Journal*.

[23] Khalifehgholi M., Shamsipour F., Ajhdarkosh H. (2013). Comparison of five diagnostic methods for Helicobacter pylori. *Iranian Journal of Microbiology*.

[24] Medina M. L., Medina M. G., Merino L. A. (2017). Correlation between virulence markers of helicobacter pylori in the oral cavity and gastric biopsies. *Arquivos de Gastroenterologia*.

[25] Garza-González E., Perez-Perez G. I., Maldonado-Garza H. J., Bosques-Padilla F. J. (2014). A review of *Helicobacter pylori* diagnosis, treatment, and methods to detect eradication. *World Journal of Gastroenterology*.

[26] Redéen S., Petersson F., Törnkrantz E., Levander H., Mårdh E., Borch K. (2011). Reliability of diagnostic tests for helicobacter pylori infection. *Gastroenterology Research and Practice*.

[27] Patel S. K., Mishra G. N., Pratap C. B., Jain A. K., Nath G. (2014). Helicobacter pylori is not eradicated after triple therapy: A nested PCR based study. *BioMed Research International*.

[28] Jalalypour F., Farajnia S., Somi M. H., Hojabri Z., Yousefzadeh R., Saeedi N. (2016). Comparative evaluation of RUT, PCR and ELISA tests for detection of infection with cytotoxigenic H. pylori. *Advanced Pharmaceutical Bulletin (APB)*.

[29] Yamaoka Y., Graham D. Y. (2014). *Helicobacter pylori* virulence and cancer pathogenesis. *Future Oncology*.

[30] Miernyk K., Morris J., Bruden D. (2011). Characterization of *Helicobacter pylori cagA* and *vacA* genotypes among Alaskans and their correlation with clinical disease. *Journal of Clinical Microbiology*.

[31] Wei G.-C., Chen J., Liu A.-Y. (2012). Prevalence of Helicobacter pylori vacA, cagA and iceA genotypes and correlation with clinical outcome. *Experimental and Therapeutic Medicine*.

[32] Kadi R. H., Halawani E. M., Abdelkader H. S. (2014). Prevalence of H. pylori strains harbouring cagA and iceA virulence genes in saudi patients with gastritis and peptic ulcer disease. *Microbiology Discovery*.

[33] Said Essa A., Alaa Eldeen Nouh M., Mohammed Ghaniam N., Graham D. Y., Said Sabry H. (2008). Prevalence of cagA in relation to clinical presentation of Helicobacter pylori infection in Egypt. *Infectious Diseases*.

[34] El-Shenawy A., Diab M., Shemis M. (2017). Detection of Helicobacter pylori vacA, cagA and iceA1 virulence genes associated with gastric diseases in Egyptian patients. *Egyptian Journal of Medical Human Genetics*.

[35] Marie M. A. M. (2012). Relationship between Helicobacter pylori virulence genes and clinical outcomes in Saudi patients. *Journal of Korean Medical Science*.

[36] Feliciano O., Gutierrez O., Valdés L. (2015). Prevalence of. *BioMed Research International*.

[37] Ben Mansour K., Fendri C., Zribi M. (2010). Prevalence of *Helicobacter pylori vacA, cagA, iceA and oipA* genotypes in Tunisian patients. *Annals of Clinical Microbiology and Antimicrobials*.

